# Extracting proficiency differences and individual characteristics in golfers' swing using single-video markerless motion analysis

**DOI:** 10.3389/fspor.2023.1272038

**Published:** 2023-11-15

**Authors:** Kota Yamamoto, Yumiko Hasegawa, Tomohiro Suzuki, Hiroo Suzuki, Hiroko Tanabe, Keisuke Fujii

**Affiliations:** ^1^ Graduate School of Informatics, Nagoya University, Nagoya, Japan; ^2^ Research Fellow of the Japan Society for the Promotion of Science, Nagoya, Japan; ^3^ Faculty of Humanities and Social Sciences, Iwate University, Morioka, Japan; ^4^ Faculty of Economics, Ryukoku University, Kyoto, Japan; ^5^ Institutes of Innovation for Future Society, Nagoya University, Nagoya, Japan

**Keywords:** golf swing, markerless motion analysis, pose estimation, object detection, proficiency, individual characteristics

## Abstract

In this study, we analyzed golfers' swing movement to extract differences in proficiency and individual characteristics using two-dimensional video data from a single camera. We conducted an experiment with 27 golfers who had a wide range of skill levels, using a 7-iron; we acquired video data with a camera on the sagittal plane. For data extraction, we used pose estimation (using HRNet) and object detection (using DeepLabCut) methods to extract human-joint and club-head data. We examined the relationship between proficiency and individual characteristics vis-à-vis forward tilt angle and club trajectory. The results showed that the stability and reproducibility of the forward tilt angle are characteristics of proficiency. Highly skilled golfers showed low variability and high reproducibility between trials in forward tilt angle. However, we found that club trajectory may not be a characteristic of proficiency but rather an individual characteristic. Club trajectory was divided roughly into clockwise rotation and counterclockwise rotation. Thus, the analysis based on video data from a single markerless camera enabled the extraction of the differences in proficiency and individual characteristics of golf swing. This suggests the usefulness of our system for simply evaluating golf swings and applying it to motor learning and coaching situations.

## 1. Introduction

Golf is a widely practiced sport, with approximately 55 million regular players worldwide (1), while many golfers train to improve their performance. Within this context, several researchers have studied the control and learning of swing movements for use in practice and instruction, as well as for injury prevention (2–5). Golf swings are highly complex because of the coordination of whole-body movements and the need to control golf clubs. This complexity has sparked considerable interest in understanding the mechanics of golfers' swing.

Sports-science researchers have studied the details of golfers' body and club movements using optical three-dimensional (3D) motion capture systems (2, 4, 6). Regarding the common characteristics of skilled players, previous analyses using 3D motion capture systems have examined the reproducibility of upper-body movements (2, 3, 7), posture at the address phase (8), swing trajectory (5, 9), club-head movement (10), the relationship between the swing plane and impact (11), swing motion, and club trajectory (12). These previous studies confirmed that a common characteristic of skilled golfers is the control of the multi-degree-of-freedom body, which reduces variability and generates a high degree of repeatability in body and club movements.

Conversely, previous studies have also revealed individual golf swing characteristics that are independent of proficiency. For example, there are three types of swing paths to the target line: inside-out, parallel, and outside-in. This difference greatly affects ball flight and is an important individual characteristic that determines performance (13, 14). Previous studies have also revealed individual differences in the patterns of the ground contact center of pressure during the swing (15, 16) and in pelvis-thorax coordination patterns (X-factor) (17). Indeed, there are individual differences in biomechanical variables, such as body and club movements during golf swings (3, 18), which emphasizes the absence of identical swings (19). The analysis of individual swing characteristics is important because it has the potential to create tailor-made practices and instruction programs for individuals. Extracting individual swing characteristics is important because it may allow the creation of personalized practice and instruction programs for individuals.

Optical motion capture systems are capable of measuring high-speed complex movements, owing to their high sampling frequencies. However, this requires considerable preparatory work, such as a large measurement location for installing an infrared camera and attaching an optical marker directly to the user's body, which is limited for outdoor use (20). Recently, swing analysis studies using cameras have used advanced image processing techniques based on computer vision methods. These studies track the movement of golf clubs and the positions of human joints to analyze subjects' motion during the swing to eliminate the disadvantages of using optical equipment (21–23). For example, Park et al. (24) obtained posture data from golf swing video recordings, while McNally et al. (25) developed a system to automatically detect continuous swing events, which are fundamental for golf swing analysis. Kim et al. (26) used both photos and videos to identify golfer metrics such as posture, swing tempo, and swing consistency during a golf swing using pose estimation. Sugimura et al. (27) developed a system to extract golf-swing defects using video data. Thus, in addition to detailed studies using motion capture systems conducted in laboratories to support golf-swing learning and instruction, golf swing research has recently been conducted using markerless video data, which are less costly to obtain. Research using video data is likely to be useful in analyzing swing data from outdoor driving ranges and rounds, as well as in obtaining data to improve performance. However, it is unclear whether single-video markerless motion analysis can effectively evaluate golfers' proficiency and individual characteristics during the swing, compared with conventional motion capture systems using reflective markers. In order to approach this problem, this research aims to apply sports science analysis for various level golfers to computer vision-related research and add practical knowledge by using single camera data. In other words, this study aims to analyze golfers' swing with a single camera to evaluate their proficiency and individual characteristics with the same degree of accuracy as a detailed analysis based on data acquired with a motion capture system. The achievement of this goal may provide insight into the development of systems that can provide support to golfers of a wide range of levels and diversity.

As described above, this study proposes a simple method for analyzing swings from markerless video data from a single camera. Particularly, we analyzed golfers' swing movement to extract differences in their proficiency and individual characteristics, revealed by 3D analysis using reflective markers from 2D data from a single camera on the sagittal plane.

This study focuses on the forward tilt angle and club trajectory, which can be analyzed using a single video camera on the sagittal plane, and examines differences in proficiency levels and individual characteristics. A forward tilt angle is necessary for accurate ball contact and is considered important in coaching situations (2, 28). As mentioned above, the swing path is directly related to the ball trajectory and is an important determinant of performance accuracy (13, 14). This study examined these two variables among golfers of various proficiency levels. Particularly, we examined the relationship between performance (ball-fall point) accuracy and the smash factor, in addition to the general score, as indicators of golfers' skill level.

This study aims to construct a simple data acquisition system and examine golfers' proficiency differences and individual movement characteristics using computer vision-based pose estimation and object detection methods for golf swing tasks. To this end, we aimed to validate a method for identifying proficiency differences and individual characteristics by calculating golf swing variables that can be analyzed with a small amount of information using two-dimensional (2D) posture data obtained from single-camera video and performance data from a trajectory measuring device.

## 2. Method

### 2.1. Participants

Twenty-seven female and male golfers (mean age: 20.5 ± 1.5 years, and mean height: 168.1 ± 7.9 cm) of a university golf club participated in the experiment (mean age of nine females: 19.8 ± 0.8 years, mean height of nine females: 161.7 ± 6.8 cm, mean age of 18 males: 20.9 ± , 1.6 years, and mean height of 18 males: 172.7 ± 5.0 cm). We recruited participants with a wide range of skill levels to examine the characteristics of proficiency. We used a questionnaire to obtain average scores of played rounds played over the past one to two years to determine each participant's golf skill level. We interviewed scores over a period of several years because the number of played rounds varied among participants. The average score of a played round is an index that reflects the overall skill of a golfer, with a lower score indicating a higher proficiency level. Handicaps are a general index, and for example, an average score of 72 means a handicap of 0, while an average score of 82 means a handicap of 10. Participants’ mean average score was 99.8 ± 25.9. The distribution of the scores was as follows: seven participants had an average of 70 (four females and three males), four had an average of 80 (all males), seven had an average of 90 (all males), and nine had an average of 100 or higher (five females and four males). The participants provided informed consent after receiving explanations about the experiment and participated in the experiment. All the experimental procedures were performed after obtaining approval from the Graduate School of Informatics of Nagoya University.

### 2.2. Procedure

Participants hit 20 balls with a 7-iron after warming up for a few minutes. We conducted the experiment at both indoor and outdoor driving ranges. The distance to the net in the direction of the ball at the indoor driving range was 3 m. Participants took a 30-second break after each trial. We instructed the participants to hit the ball as they normally would using a 7-iron, and to hit 20 balls. In this experiment, we analyzed all trials for each participant. We recorded the swing motion data with a camera (SONY RX100M7, 240 Hz, 1,824 × 616 dpi). The camera was positioned 3.5 m behind the ball in the targetline direction and in the sagittal plane of the golfer's body, so that the whole body and the club were within the angle of view of the camera. In addition, a trajectory measuring device (Skytrak, GPRO Co., Japan) was used to acquire data such as distance, ball velocity, club velocity, and left-right deviation from target line of the arrival position.

### 2.3. Data extraction

#### 2.3.1. Pose estimation for body movement with HRNet

**Figure 1:**
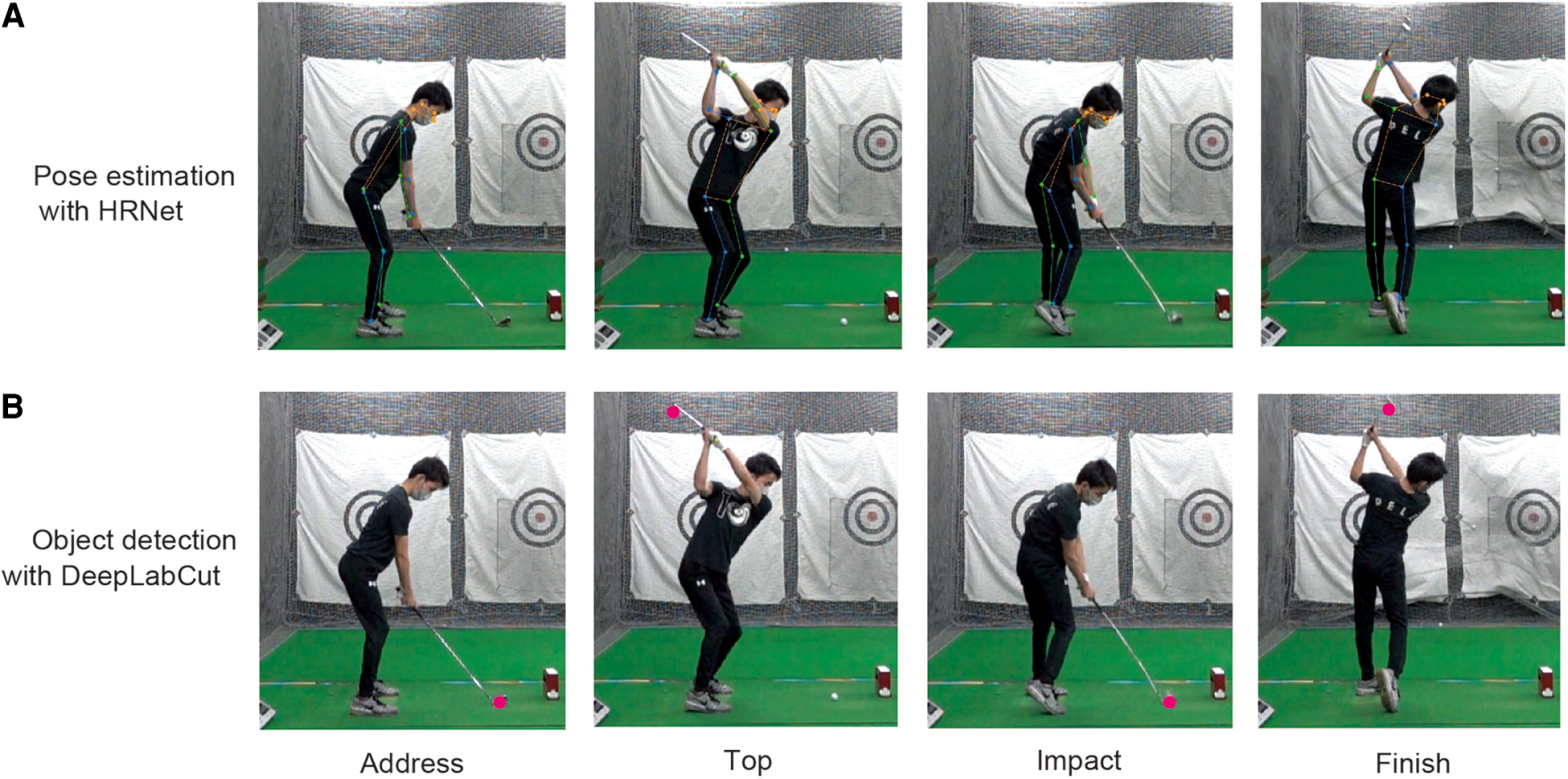
Pose estimation and object detection of swing movement. This figure shows an example of pose estimation and object detection based on 2D pose data. The panel (**A**) shows examples of pose estimation for address, top, impact, and finish swing events for 17 joint points of the body using HRNet. The panel (**B**) shows the detection results of club heads (red markers) using DeepLabCut at each event. We used HRNet-based pose estimation data for the body data and DeepLabCut based object detection data for the club head data.

Next, the joint data for the swinging motion were obtained from the video data using a computer vision method. We used HRNet (29), a framework for pose estimation in computer vision methods, to estimate the joint points of the body using the video data obtained as the input (Figure 1A).

There are two types of pose estimation models: a top-down model that estimates the key points separately after estimating the positions of a person in the image, and a bottom-up model that estimates all key points in the image and then groups them for a person. HRNet is a top-down pose estimation model. We used a model called HRNet w48 to estimate joint positions. This method used video data as input and trained general human pose data to estimate 17 key points: the nose, left and right eyes, ears, shoulders, elbows, wrists, hips, knees, and ankles. This method requires no special annotation or model creation phase. We then obtained the position data of the vertical and horizontal pixels as the output.

#### 2.3.2. Object detection for club movement with DeepLabCut

Next, we performed object detection using video data to obtain club-head position data during the swing. DeepLabCut is a video-analysis library for object detection that uses a graphical user interface to label and automatically track human and animal body parts using deep neural networks (30).

First, a pretrained model was created using 27 video datasets, with one trial for each participant. We used video data on the sagittal plane as the training data and annotated the positions of club head for 30 frames in each video image.

As preliminary estimation tests showed that the accuracy of the estimation of the club head near impact tended to decrease, the images used for annotation were extracted manually, focusing on the phase from address to impact. After annotation, we used the ResNet-50 model (31) to train the training data with 200,000 iterations. The data were then used to correct for mis-estimated points, and the model was trained again for 120,000 iterations. Finally, we estimated all 20 trials of each participant's video to obtain the body and club-head data (Figure 1B). The data obtained from the pose estimation and object detection were smoothed using a second-order Butterworth filter (6 Hz).

#### 2.3.3. Swing event detection

**Figure 2:**
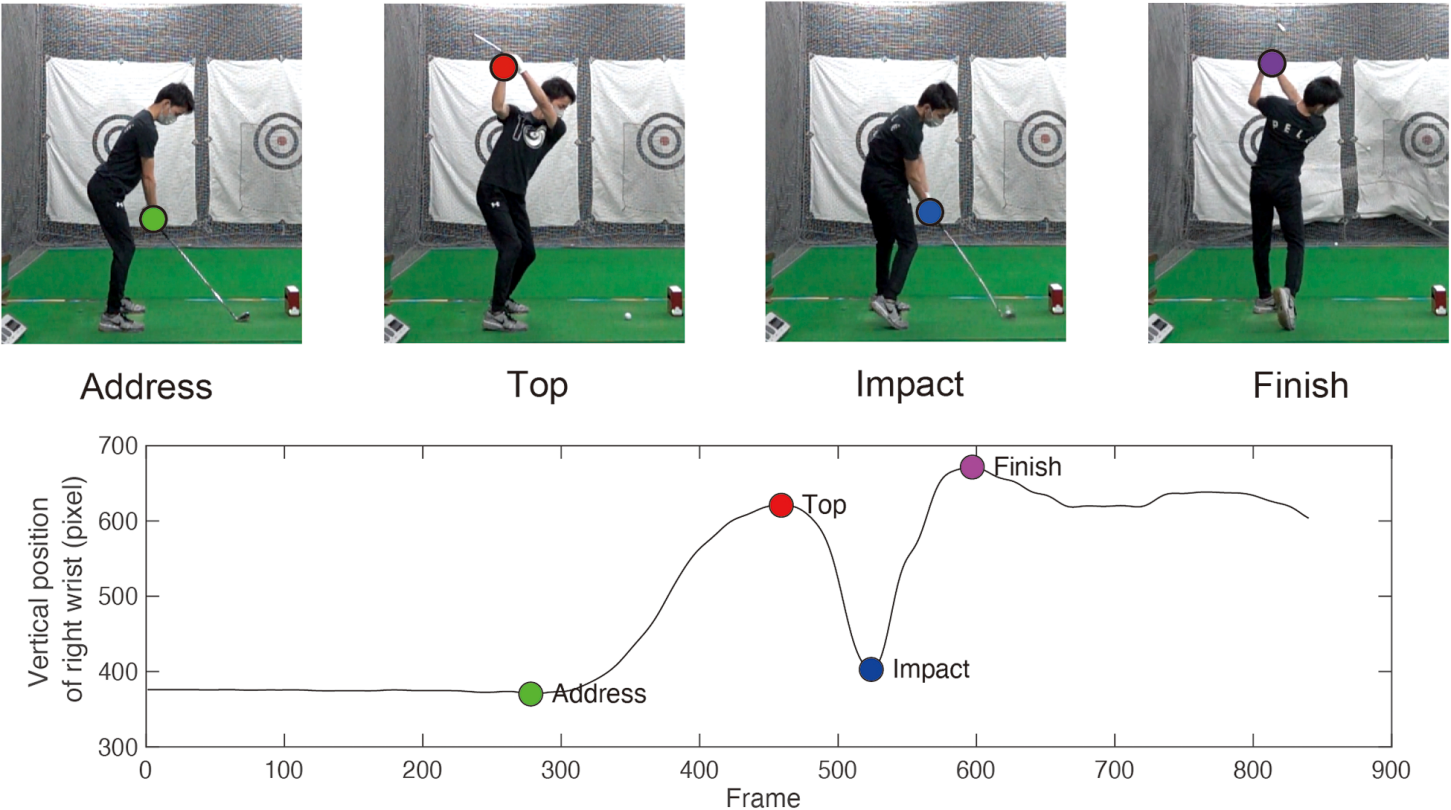
Swing event detection. Significant events during the swing were calculated from body data obtained by pose estimation and each event was calculated based on the vertical position of the right wrist on the sagittal plane. The upper panel shows a plot of the right wrist's position at each event. The lower panel shows time-series data of the right wrist's position during a single swing. The markers in the upper and lower figures indicate the wrist position at each event. The green marker indicates the event at address before the swing, the red marker indicates the event at the top switching between backswing and downswing, the blue marker indicates the event at impact, and the purple marker indicates the event at the finish after hitting the ball.

We detected the address, top, impact, and finish events of the golf swing by using a custom-written software in MATLAB (R2022a; MathWorks, Natick, MA, USA). We used the position data of the right wrist on the sagittal plane to detect the events (Figure 2). For the address event, we detected the rising frame of the right wrist position. We calculated the velocity from the wrist position and detected the frame when the velocity exceeded a certain threshold value. We then defined the point at which 30 frames (0.125 sec) back from that rising point as the address event.

The maximum value of the position was detected for the top event. For the impact event, we detected the frame in which the wrist reached its minimum value after reaching the top; for the finish event, we detected the maximum wrist position after the impact. In addition to these four events, we detected events when the wrist exceeded the shoulder position during the backswing and when the wrist fell below the shoulder position during the downswing for swing trajectory analysis.

### 2.4. Analysis

After preprocessing the data, we analyzed the golf swings to examine proficiency differences and individual characteristics. In this study, the forward tilt angle and swing trajectory were calculated. These two variables can be obtained from video data in the sagittal plane and are important variables in golf (14). These two variables were examined in terms of proficiency differences and individual differences. All data analyses were carried out in MATLAB.

#### 2.4.1. Forward tilt angle

**Figure 3:**
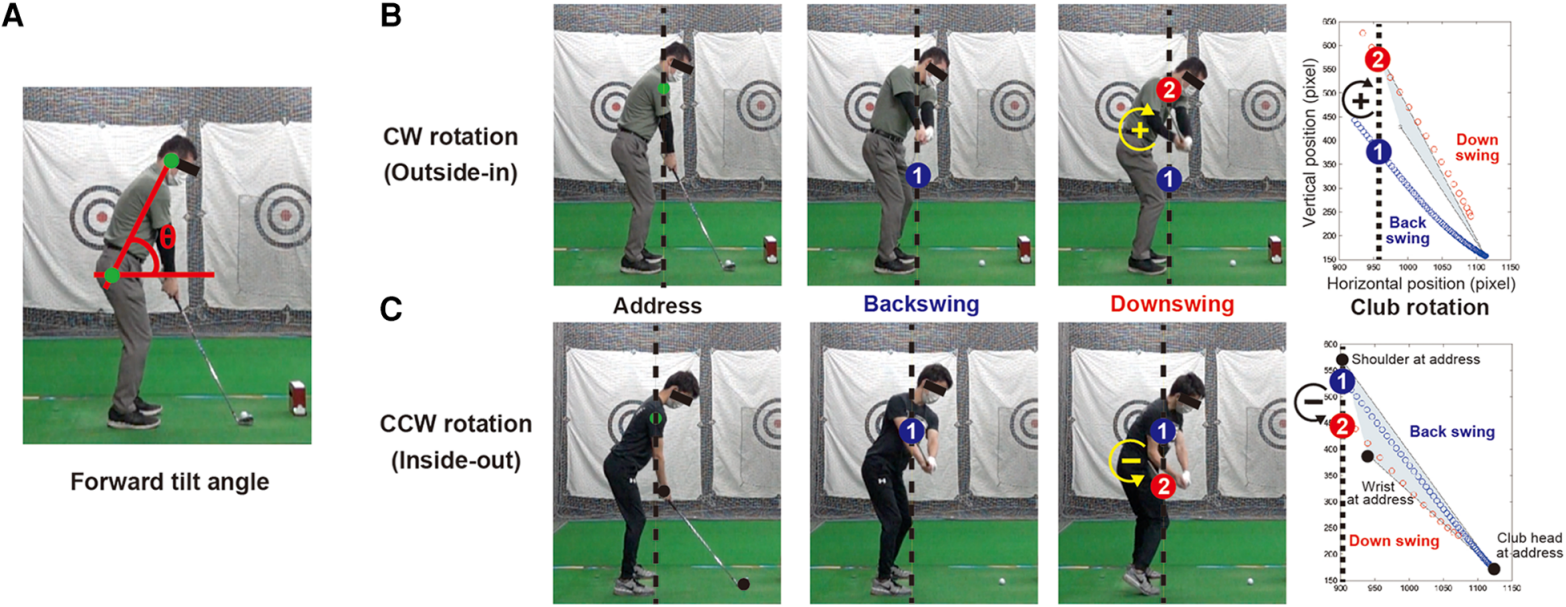
Quantification of swing trajectory by using rotation between backswing and downswing. The panel (**A**) shows the definition of the forward tilt angle, we calculated the angle between the right ear and right hip vectors on the sagittal plane and the horizontal line. The panel (**B**) indicates a participant of the clockwise (CW) rotation and the panel (C) indicates a participant of the counterclockwise (CCW) rotation. Swing images during address, backswing, and downswing are shown on the left, center, and right columns, respectively. The dash line indicates the position of the right shoulder at address, while the blue circle with the number 1 indicates the position past the right shoulder line during the backswing. The red circle with the number 2 indicates the position past the right shoulder line during the downswing. The rightmost plot shows the club position during the backswing and downswing and the swing plane enclosed by the right shoulder and right wrist at address.

First, for the forward tilt angle, we calculated the angle between the right ear and right hip vectors on the sagittal plane and the horizontal line in the phase from the address to impact (Figure 3A). After calculating the mean and standard deviation of the forward tilt angle for the phase from address to impact, we calculated the following variables: (1) the average of all 20 trials of the forward tilt angle (mean angle), (2) the average of the SD of the forward tilt angle over all trials (mean SD), (3) the SD of all trials of the average of the forward tilt angle (SD of mean angle), and (4) the SD of all trials of the SD of the forward tilt angle (SD of SD). The first variable is the average tilt angle of all trials for each participant. The second variable is the average variability of forward tilt angle during a single swing and the third indicates the extent to which the forward tilt angle varies from swing to swing. The fourth variable indicates the extent to which the forward tilt angle varies from swing to swing.

#### 2.4.2. Club rotation

In this study, we analyzed the club trajectory during the swing phase using 2D data on the sagittal plane using the following method. Swing trajectories were examined for backswing and downswing club rotations based on the trajectory of the club head movements in the sagittal plane. This rotation implies a difference between the route of club pull-up during backswing and the route of club down during downswing.

Typical examples of the two swing trajectories observed in the experiment are shown in Figures 3B,C. The blue plots show the points of the club head during the backswing (from the address until the right wrist crosses the right shoulder). The red plot shows each point on the club head during downswing (until the right wrist crosses the right shoulder). The gray area surrounded by the black plot and lines represents the swing plane at the address. The upper line shows the line connecting the club head and right shoulder at the address (swing plane), and the lower line shows the line connecting the club head and right wrist at the address (shaft plane).

The example in Figure 3B shows a club trajectory where the downswing comes down from above compared to the backswing. From the viewpoint of the sagittal plane, the club rotates clockwise from the backswing to the downswing. The example in the Figure 3C shows a club trajectory in which the downswing descends from below compared to the backswing, and from a sagittal viewpoint, the rotation is counterclockwise from the backswing to the downswing. Generally, the swing path on the left side of the figure is known to be an outside-in trajectory with the club head entering from the outside and exiting from the inside with respect to the ball's direction of travel, whereas the swing path on the right side of the figure is known to be an inside-out trajectory with the club head entering from the inside and exiting from the outside (14).

We quantified the pattern of the club trajectory characteristics on the sagittal plane, as shown in Figures 3B,C. The vertical passing positions during backswing and downswing were compared with respect to the shoulder position at the address. Figure 3B shows that the backswing passes through the right shoulder at address, while the downswing passes through the right shoulder at address for the participant showing clockwise club rotation (CW rotation). Figure 3C shows each phase of the participant showing the counterclockwise club rotation (CCW rotation) described above. In principle, the CW rotation has a steep angle of attack, whereas the CCW rotation has a shallow angle at ball impact, which affects the direction and distance of the hit ball.

We calculated the differences between the vertical position of the point passing through the right shoulder at address during backswing and that during downswing as the club rotation variable. In the case of clockwise rotation, where the downswing passes through the upper side, as shown in the upper panel of Figure 3, the club rotation takes a positive value, while a larger value indicates that the golfer is backswinging from the upper side. However, in the case of counterclockwise rotation, where the downswing passes below, as in the golfer below, the value of the club rotation is negative. We evaluated the direction of club rotation by using numerical values and examined the relationship with the level of proficiency.

#### 2.4.3. Proficiency level

We used the golfers' average scores as indicators of their proficiency. Additionally, we calculated the smash factor, which is related to swing efficiency. The smash factor indicates the ratio of ball speed to club speed and is an indicator of how much club speed is being transferred to the ball. The accuracy of the angle of the ball landing position was evaluated by calculating the angle between the golfer's position and the vector of the landing point and the target line, and the average of the absolute values was used as the index. In other words, if 20 balls landed on the target line, this index value would be as close to zero as possible.

## 3. Results

### 3.1. Analysis of the forward tilt angle

#### 3.1.1. Variability of forward tilt angle during the swing

**Figure 4:**
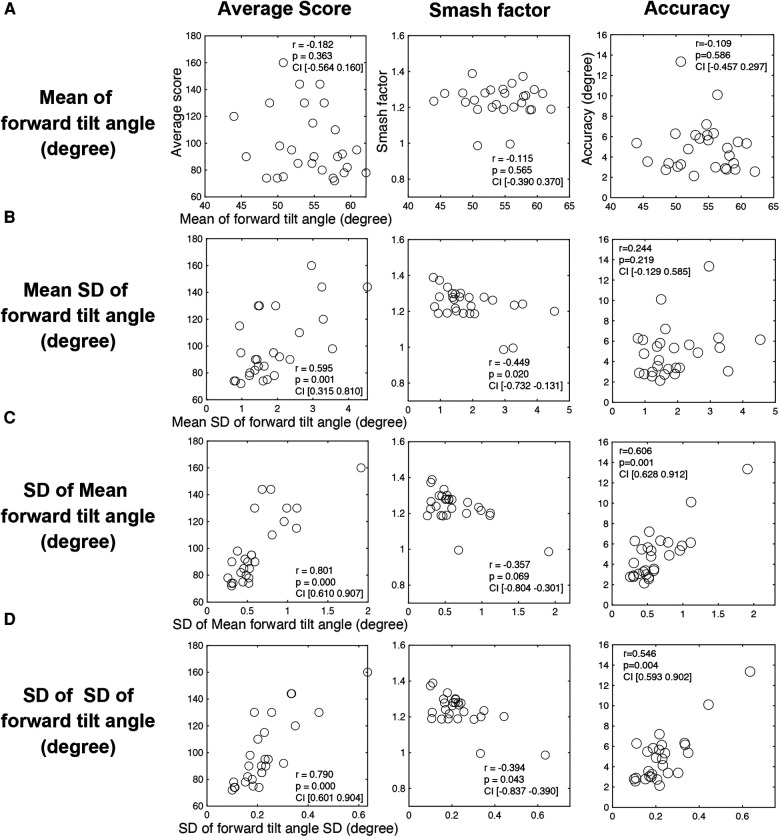
Correlation between proficiency indices and indices of forward tilt angle during swing movement. The columns indicate the proficiency indices used in the correlation analysis, while the rows indicate the variables of forward tilt angle used in the analysis. Pearson's correlation coefficient (r), *p*-value, and 95% confidence interval are noted in each plot.

First, we examined each participant's average forward tilt angle during the swing phase with the 7-iron. We performed a correlation analysis among the average of all 20 trials of the forward tilt angle from address to impact, the average score, the smash factor, and the variance of the fall position. The results showed that none of the correlations were significant between the mean forward tilt angle and the proficiency indices (average score: *r* = −0.182, n.s., 95% *CI* [−0.564, 0.160]; smash factor: *r* = 0.115, n.s., 95% *CI* [−0.390, 0.370]; accuracy: *r* = 0.109, n.s., 95% *CI* [−0.457, 0.297], shown in the Figure 4A). Next, we examined the forward tilt angle of the upper body during the swing. We performed a correlation analysis of the three variables of the forward tilt angle described above with the average score of the proficiency index, the smash factor, and the angular of the ball's landing position.

Correlation analysis of the mean SD of the forward tilt angle with the average score revealed a moderate correlation between the average score [*r* = 0.595, *p* < 0.01, 95% *CI* (0.315, 0.810)] and smash factor [*r* = 0.449, *p* < 0.05, 95% *CI* (−0.732, −0.131)]. The results showed that the variability in the forward tilt angle during the swing was smaller for golfers with better average scores and those with better smash factors (shown in the Figure 4B).

#### 3.1.2. Reproducibility of the forward tilt angle during the swing

Next, we examined the correlation between the SD of all trials of the mean value of the forward tilt angle from the address to the impact and each proficiency index. The results showed a significant correlation between the SD of all trials of the mean anterior tilt angle and the average score [*r* = 0.801, *p* < 0.01, 95% *CI* (0.610, 0.907)] and accuracy [*r* = 0.606, *p* < 0.01, 95% *CI* (0.628, 0.912)] (shown in the Figure 4C). The results revealed that golfers with better average scores and higher accuracy had better reproducibility of the forward tilt angle between swing trials.

#### 3.1.3. Reproducibility of variability in the forward tilt angle during the swing

Next, we examined the correlation between the all trials SD of the SD of the forward tilt angle from address to impact and each proficiency index. The results showed a significant correlation between the SD of all trials of the forward tilt angle and the average score [*r* = 0.790, *p* < 0.01, 95% *CI* (0.601, 0.904)], smash factor [*r* = 0.394, *p* < 0.05, 95% *CI* (−0.837, −0.390)], and accuracy [*r* = 0.546, *p* < 0.01, 95% *CI* (0.593, 0.902)] (shown in the Figure 4D). The results showed that participants with higher average scores, accuracy, and smash factors had smaller trial-to-trial variability in mean forward tilt angle during the swing. In other words, more proficient golfers showed a higher reproducibility of variability in the forward tilt angle between swings. The results of the within- and between-trial variability of the forward lean angle revealed that proficient subjects were able to maintain a forward lean angle with some degree of variability. Additionally, it was found that the adept had a high degree of reproducibility in the magnitude and variability of fluctuations.

### 3.2. Analysis of swing trajectory on the sagittal plane

**Figure 5:**
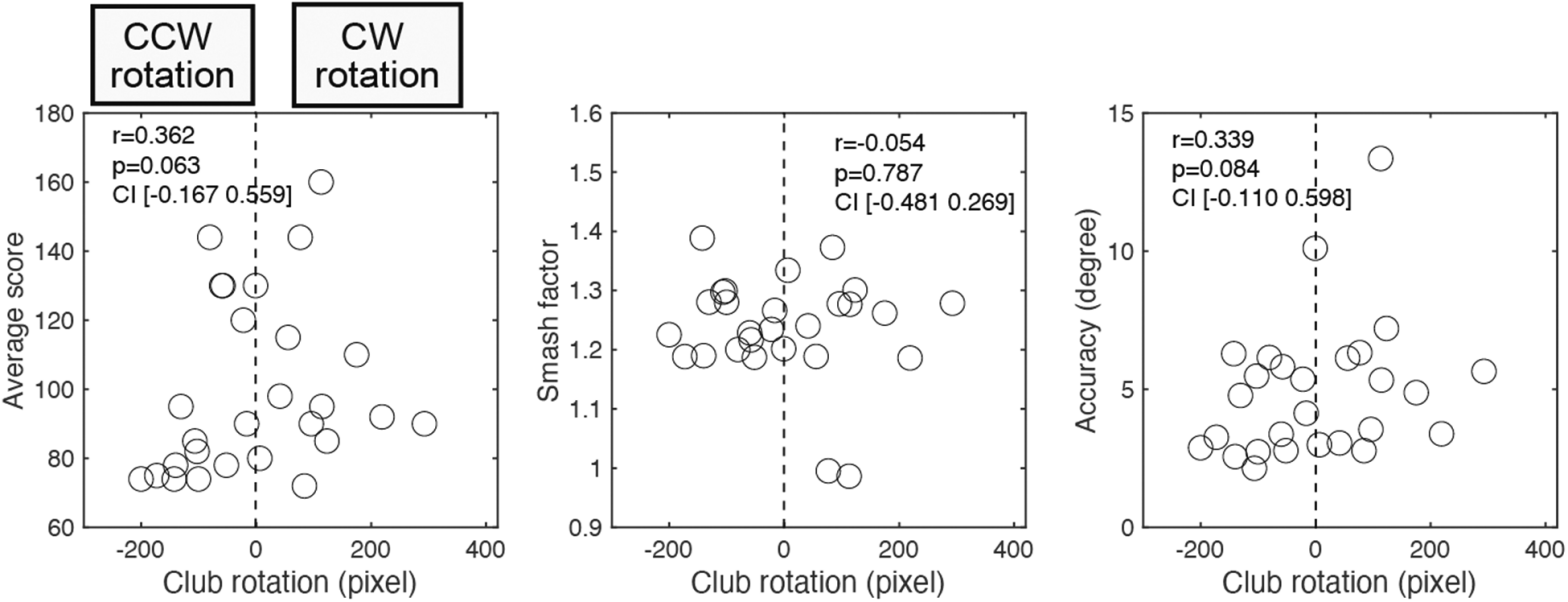
Correlation between proficiency indices and club rotation. The vertical axis indicates the average score, smash factor, and accuracy of the fall position, while the horizontal axis shows the value of club rotation. For club rotation on the horizontal axis, positive values refer to CW rotation and negative values to CCW rotation. Pearson's correlation coefficient (r), *p*-value, and 95% confidence interval are noted in each plot.

Next, we examined proficiency and individual characteristics in the swing trajectory by examining the relationship between club rotation and proficiency variables (Figure 5). We performed a correlation analysis between the club rotation value and each of the proficiency indices: the average score, smash factor, and accuracy index. The results showed no significant correlation between the club rotation value and average score [*r* = 0.362, n.s., 95% *CI* (−0.167, 0.559)], smash factor [*r* = 0.054, n.s., 95% *CI* (−0.481, 0.269)], and accuracy [*r* = 0.339, n.s., 95% *CI* (−0.110, 0.598)]. These results indicate that neither the club trajectory pattern nor its magnitude were associated with proficiency. In other words, it is possible that the movement pattern of how a club is raised and lowered does not indicate differences in proficiency; rather, it is unique to each golfer.

## 4. Discussion

### 4.1. Swing analysis using computer vision methods

This study examined the trade-off between a small amount of information data using computer-vision-based pose estimation and object detection methods for a golf-swing task, and the possibility of assessing movement proficiency and extracting individual characteristics. In this study, we used HRNet for pose estimation at the joint points of the body and DeepLabCut for club-head object detection. The 2D body and club data obtained allowed the calculation of important golf variables. For example, the average forward tilt angle obtained in this study ranged from 45 ° to 60 °, relative to the horizontal line. The results for the forward tilt angle in this study did not differ significantly from the results obtained with a lumbar-motion monitor in experiments using a 7-iron (32).

Because the golf swing is a 3D motion involving the rotation and twisting of the body, the method used in this study is inferior with respect to accuracy, compared with 3D motion analysis. The model called HRNet w48 which was used for the posture estimation in this study has been reported to have a high average precision and high estimation accuracy among many models (29). In addition, the process of checking the accuracy and modifying the model for object detection using DeepLabCut in combination with the inferred data and video data was used to create a model that can be estimated with high accuracy. However, the accuracy of the posture estimation data used in this study will require future experiments to verify the consistency with 3D motion capture data. Although there are limits on accuracy of data acquisition, the results of this study show that it is possible to evaluate the proficiency of a golfer's swing even with 2D information obtained from a single camera. Regarding the club trajectory results, we extracted club trajectories on the sagittal plane and were able to detect individual characteristics regarding swing switching. This also suggests the possibility of extracting the characteristics of each golfer from the trajectory of the club head in two dimensions using markerless pose estimation. However, many issues remain, including verification of the accuracy of pose estimation and object detection, analysis of additional golfer data, and implementation in applications.

### 4.2. Proficiency differences in the golf swing

In this study, we examined changes in the forward tilt angle on the sagittal plane and club trajectory during the swing in terms of differences in proficiency. Only forward tilt angle was significantly correlated with proficiency. Particularly, the results obtained in this study showed a significant correlation between the stability of the average forward tilt angle during the swing and the repeatability and variability of the angle across 20 trials. However, the average forward tilt angle was not associated with proficiency. This suggests that it is not important for the upper body to have a particular forward tilt angle during the 7-iron swing, but rather that one factor in proficiency is being able to swing at a similar angle with a similar variation in angle repeatedly, reducing the variability during the swing.

Regarding the stability of the average forward tilt angle during the swing, the results of this study demonstrate the importance of a constant forward tilt angle. These results confirm the importance of maintaining a forward tilt during the swing for performance stability, as suggested by Chu et al. (2). An important factor for consistent performance in golf swing tasks has been shown to be a strategy for maintaining constant upper body variability in order to make accurate contact with the ball (28). A correlation between the degree of proficiency and reproducibility across trials was also observed, suggesting that one factor of proficiency is not only the ability to maintain a constant upper body angle during the swing but also the ability to repeat similar movements to produce a consistent performance. Particularly, the stability and reproducibility of the forward tilt angle were confirmed to be related to the smash factor, ball-direction accuracy, and average score. This indicates the importance of maintaining posture during the swing to correctly contact the club face, efficiently transfer swing speed to the ball, and contact the ball in the correct orientation and direction. This suggests that changes in the forward tilt angle during the swing can be confirmed using a single camera on the sagittal plane and can be used to evaluate proficiency. Further, no correlation was found between the average angle and the proficiency index, whereas a correlation with the stability and reproducibility of the angle was confirmed, suggesting that it is important to aim for consistency and reproducibility of similar movements repeatedly, rather than learning aimed at a specific angle.

### 4.3. Individual characteristics in the golf swing

Next, we examined club trajectories in the sagittal plane. In this study, we examined the difference between the club trajectory during backswing and that during downswing to extract features from 2D data on the sagittal plane for the club trajectory. We defined CW rotation as a trajectory that passes lower on the downswing than on the backswing; conversely, CCW rotation was defined as a trajectory that passes higher on the downswing than on the backswing. This difference in club rotation is generally related to the difference in swing trajectory and angle of ball impact in the direction lateral to the target line on the delivery plane. There were three types of swing paths to the target line: inside-out, parallel, and outside-in. This difference greatly affects ball flight and is an important individual characteristic that determines performance (13, 14).

Based on the data obtained in this study, we examined the relationship between golfers' club rotation pattern and lateral launch angle obtained using a trajectory-measuring device. We found a significant correlation between rotation and lateral launch angle (*r* = −0.57, *p* < 0.05). The results show that in the case of CW rotation (downswing passes through the upper side of the club, rather than the backswing), the lateral launch direction was to the left of the target line, while the club had an outside-in trajectory passing from the outside to the inside. Conversely, in the case of CCW rotation (downswing passing below the backswing), the left lateral launch direction was to the right of the target line, while the club had an inside-out trajectory from the inside to the outside. This indicates that the variables of club rotation, which can be easily calculated from the data on the sagittal plane analyzed in this study, may be used, as well as the types of club and swing trajectories from conventional 3D analysis.

The results of this study did not suggest a correlation between individual club rotation characteristics and proficiency. In terms of club rotation characteristics, 12 golfers exhibited CW rotation and 15 exhibited CCW rotation. This indicates that there were large individual differences in the way clubs moved and there was no pattern of convergence in the process of proficiency. However, a correlation (*r* = 0.52, *p* < 0.01) was found between the average score and club rotation when only participants with average scores below 100 were included. This confirms the tendency of more proficient golfers to exhibit a CCW rotation, in which the downswing trajectory passes below the backswing trajectory. In other words, although no relationship with proficiency was found in the analysis of participants as a whole, it is possible that convergence to the CCW rotation pattern could be observed in golfers with higher than intermediate levels of proficiency. This point should be verified in future research including more proficient golfers.

## 5. Conclusion

This study aimed at the examination of simple data acquisition system using computer vision-based pose estimation and object-detection methods for a golf-swing task, and the possibility of assessing movement proficiency and extracting individual characteristics. Specifically, we assumed a smartphone application for practice and instruction and aimed to evaluate golf swing proficiency and identify variables that determine individual characteristics from simple 2D pose data obtained from a single camera. Consequently, the 2D pose data extracted from a single camera using computer-vision techniques confirmed the characteristics of golfers’ forward tilt angle, which is a feature of proficiency. Additionally, we identified the individual characteristics of swing trajectory. In other words, the analysis based on video data from a single markerless camera enabled the extraction of the proficiency and individual characteristics of participants' golf swing. This highlights the usefulness of our system for simply evaluating golf swings and applying it to motor learning and coaching situations.

## Data Availability

The raw data supporting the conclusions of this article will be made available by the authors, without undue reservation.
